# Research and Remedies

**Published:** 1912-12-14

**Authors:** 


### RESEARCH AND REMEDIES.

### An Investigation of Soap.

The effects of various soaps upon the skin has been examined by Dr. Frederick Gardiner, who issues his results in the *Edinburgh Medical Journal*. Chemical, bacteriological, and clinical tests were employed, including some experiments upon himself which can hardly have been pleasant. All soaps, he finds, must be from their chemical composition irritant to the normal skin; the effect varies with the individual, and is greater in senile and diseased skins. Cotton-seed oil and rancid fats are nowadays much used in the manufacture of cheap soaps and probably account for their irritant effects. The bacteriological power of soap is *nil*; and this applies not only to ordinary soaps, but also to those impregnated with disinfectants or antiseptics. Sulphur, ichthyol, and similar substances may have some effects upon the glands and blood vessels of the skin, but carbolic and other antiseptics merely increase irritation. There is no scientific basis, Dr. Gardiner says, for the addition of extra fat to soap; the only virtue in a superfatted soap is that the higher price at which it is sold generally means that the makers can afford to use very pure materials. Rosin is less detrimental than is generally supposed as an ingredient of soap.

### Choroidal Tubercles in Tuberculous Meningitis.

The diagnosis of tuberculous meningitis has been very greatly facilitated and expedited during recent years by the adoption of lumbar puncture, with examination of the cerebro-spinal fluid for tubercle bacilli. In consequence the diagnostic importance of choroidal tubercle and of frequent ophthalmoscopic examinations for its detection is perhaps less than it was a few years ago. Nevertheless there is still a fair percentage of cases in which the discovery of choroidal tubercles will clinch the diagnosis more quickly than any other method of investigation. Carpenter and Stephenson long ago published their statistics showing that this lesion was observed by them in 50 per cent. of their cases; and they suggested that frequently repeated observations might raise the percentage much higher, even to 100. Marple, of New York, examined one hundred and twenty-eight cases in four years, and found tubercles in only 5 per cent., and other American observers reported similar figures. Determined to get to the bottom of this marked discrepancy, he made arrangements to have the fundus oculi of all his patients systematically and frequently searched by skilled observers. This resulted in a great change: in thirteen children, suffering from undoubted tuberculous meningitis who were thus examined, every single one revealed lesions in the fundus at some time or other before death. In some cases two or three fresh tubercles would appear within twelve hours; and in one case his house physician found a tubercle four hours after Marple had pronounced the fundus normal. He concludes that Carpenter and Stephenson are right, and that the percentage in which this sign is observed depends on the completeness of the observations. He further notes as a minor point that in all forms of choroiditis other than tuberculous some degree of exudate upon Descemet's membrane will be present. The paper in which he embodies these interesting researches is published in the *Ophthalmoscope*.

### The Transplantation Treatment of Addison's Disease.

The disease which Addison was the first to describe accurately, now known by his name, is due to destruction of the suprarenal bodies, usually by tuberculosis. It is almost uniformly fatal, whenever it has progressed so far as to render diagnosis probable. Treatment by all kinds of drugs serves but to postpone, as a rule, the inevitable result; even the administration of suprarenal extract fails to do permanent good, though it may ameliorate the symptoms for a time. From Australia comes the report of an extraordinary case, in which, after all other measures had failed, transplantation of the suprarenal gland from one body to another has been practised, and so far, apparently, with success. The patient was a woman of thirty-five, who came under care for attacks of vomiting combined with anæmia. An exploratory operation was performed, and some adhesions found about the appendix, which was removed. Soon afterwards pigmentation and low blood-pressure were noticed, and the diagnosis of Addison's disease was confirmed by a positive von Pirquet reaction. Tuberculin treatment was tried, also iron, arsenic, and suprarenal extract, with temporary success; but soon the patient began to go downhill again. It was decided to attempt a transplantation experiment. One day, in the same hospital, a man died from heart disease; he was free from syphilis and tuberculosis. Immediately after death the right kidney was removed, with full aseptic precautions, and the adrenal was immediately detached and placed in normal saline solution. The adrenal was then freed from fat and bisected with a sharp knife. Meanwhile, under local anæsthesia, incisions had been made over the lower ends of the recti abdominis muscles of the woman patient, and the muscle sheaths opened. Half of the gland was then buried in each muscle, and the wound sutured up. The patient at the time of the operation was in a serious condition, and for four days she exhibited little improvement. From that time onwards she improved rapidly, and within a fortnight was better than she had been for a year. Four months after the operation she was still well, and able to do light household duties. Pigmentation was diminishing, vomiting had ceased, and the weight had increased a stone. The operator, Dr. Morton, who describes the case in the *Australian Medical Journal*, admits that time has yet to show whether this case will be a permanent success; but he promises to report progress, whether the future history be good or bad.

